# Deciphering the molecular heterogeneity of intermediate- and (very-)high-risk non–muscle-invasive bladder cancer using multi-layered *–omics* studies

**DOI:** 10.3389/fonc.2024.1424293

**Published:** 2024-10-21

**Authors:** Murat Akand, Tatjana Jatsenko, Tim Muilwijk, Thomas Gevaert, Steven Joniau, Frank Van der Aa

**Affiliations:** ^1^ Department of Urology, University Hospitals Leuven, Leuven, Belgium; ^2^ Laboratory of Experimental Urology, Urogenital, Abdominal and Plastic Surgery, Department of Development and Regeneration, KU Leuven, Leuven, Belgium; ^3^ Laboratory for Cytogenetics and Genome Research, KU Leuven, Leuven, Belgium; ^4^ Center for Human Genetics, University Hospitals Leuven, Leuven, Belgium; ^5^ Department of Pathology, AZ Klina, Brasschaat, Belgium

**Keywords:** bladder cancer, recurrence, progression, non-muscle-invasive, -*omics*, genomics, epigenetics, transcriptomics

## Abstract

Bladder cancer (BC) is the most common malignancy of the urinary tract. About 75% of all BC patients present with non-muscle-invasive BC (NMIBC), of which up to 70% will recur, and 15% will progress in stage and grade. As the recurrence and progression rates of NMIBC are strongly associated with some clinical and pathological factors, several risk stratification models have been developed to individually predict the short- and long-term risks of disease recurrence and progression. The NMIBC patients are stratified into four risk groups as low-, intermediate-, high-risk, and very high-risk by the European Association of Urology (EAU). Significant heterogeneity in terms of oncological outcomes and prognosis has been observed among NMIBC patients within the same EAU risk group, which has been partly attributed to the intrinsic heterogeneity of BC at the molecular level. Currently, we have a poor understanding of how to distinguish intermediate- and (very-)high-risk NMIBC with poor outcomes from those with a more benign disease course and lack predictive/prognostic tools that can specifically stratify them according to their pathologic and molecular properties. There is an unmet need for developing a more accurate scoring system that considers the treatment they receive after TURBT to enable their better stratification for further follow-up regimens and treatment selection, based also on a better response prediction to the treatment. Based on these facts, by employing a multi-layered *–omics* (namely, genomics, epigenetics, transcriptomics, proteomics, lipidomics, metabolomics) and immunohistopathology approach, we hypothesize to decipher molecular heterogeneity of intermediate- and (very-)high-risk NMIBC and to better stratify the patients with this disease. A combination of different –*omics* will provide a more detailed and multi-dimensional characterization of the tumor and represent the broad spectrum of NMIBC phenotypes, which will help to decipher the molecular heterogeneity of intermediate- and (very-)high-risk NMIBC. We think that this combinatorial multi*-omics* approach has the potential to improve the prediction of recurrence and progression with higher precision and to develop a molecular feature-based algorithm for stratifying the patients properly and guiding their therapeutic interventions in a personalized manner.

## 1 Introduction and background

Bladder cancer (BC) is the most common malignancy of the urinary tract and is the sixth most commonly diagnosed form of cancer in Europe, while it ranked ninth worldwide (1, 2). Moreover, the estimated number of new cases will almost double from 2022 to 2050 (3). Although BC is approximately four times more commonly seen in males, females are more frequently diagnosed with advanced-stage disease when compared to males in the same age group (4). This gender discrepancy is partially attributed to gender differences in smoking, which can also explain the increasing incidence of BC in women in developed countries. Tobacco smoking is by far the principal risk factor for the development of BC, accounting for approximately 50-65% of new cases each year. Occupational exposure to carcinogens (including aromatic amines, polycyclic aromatic hydrocarbons, and chlorinated hydrocarbons) is the second most frequent preventable risk factor for BC in industrialized countries. These compounds are commonly found in industries where dyes, paint, metal, rubber, and petroleum products are produced (5, 6). Apart from a history of pelvic radiotherapy, the other risk factors for BC, for which the International Agency for Research on Cancer (IARC) has reported sufficient evidence, are environmental exposures (arsenic, X or gamma radiation), medications (cyclophosphamide, chlornaphazine), opium consumption, and Schistosoma infection (7). Moreover, candidate association studies have unveiled that the gene polymorphisms of N-acetyltransferase (*NAT-2*) and glutathione S transferases (*GSTM1* and *GSTT1*) (detoxifying arylamines and polycyclic hydrocarbons), and of arsenic (+3 oxidation state) methyltransferase (*AS3MT*) are associated with a higher risk of BC (6, 8). At present, Lynch syndrome, caused by germline mutations in DNA mismatch repair (MMR) genes (*MSH2*, *MSH6*, *MLH1*, *PSM2*, and *EPCAM*), remains the only identified hereditary cancer syndrome associated with a higher BC risk (8).

More than 90% of BCs in Europe and North America are urothelial carcinomas (UC), derived from the urothelium. Confirmation of the diagnosis and clinical staging is performed by transurethral resection of the bladder tumor (TURBT). At initial presentation, 25% of cases are diagnosed with muscle-invasive BC (MIBC). These patients have aggressive disease, as approximately one-third have undetected metastases, while 25% have lymph node involvement (9). Treatment modalities are well defined by international guidelines, with aggressive combination treatments, such as radical cystectomy with (neo-)adjuvant chemotherapy, to be recommended (9). The remaining 75% of BC patients present with non–muscle-invasive BC (NMIBC), disease confined to the mucosa (stage Ta, carcinoma *in situ* [CIS]) or submucosa (stage T1). Up to 70% of the NMIBC cases will recur, and 15% will progress in stage and grade (10). Therefore, NMIBC patients are scheduled to undergo frequent monitoring, currently based on cystoscopy and cytology, which makes BC one of the costliest of all cancers to manage (11).

The recurrence and progression rates of NMIBC are strongly associated with several clinical and pathological factors. To individually predict the short- and long-term risks of disease recurrence and progression, the European Organization for Research and Treatment of Cancer (EORTC) Genito-Urinary Cancer Group (GUCG) has developed a risk calculator consisting of a scoring system and risk tables (12). The EORTC risk calculator is the result of a *post-hoc* statistical analysis of 2596 patients from seven separate prospective trials with 291 to 517 included patients. These patients, treated between 1979 and 1989, were categorized by the old (pre-2004) World Health Organization (WHO) grading system. Because only a minority of patients (n=171) in the EORTC cohort were treated with bacillus Calmette-Guérin (BCG), and none of them received maintenance treatment (which is now considered mandatory for at least 12 months to lead to effect), the Spanish CUETO (Club Urologico Español de Tratamiento Oncologico) consortium developed another risk stratification model based on a total of 1062 patients treated with BCG between February 1990 and May 1999 in 4 prospective trials (13). Both risk calculators have poor discrimination for prognostic outcomes in external validation (14, 15). In a Brazilian cohort, the discriminative ability of the EORTC model overestimated the short- and long-term progression rates, especially in high-risk patients (14). Another retrospective multicentric study demonstrated that both the EORTC and the CUETO risk calculators overestimated the risk of disease recurrence and progression in high-risk patients. Moreover, it was observed that these overestimations remained in the BCG-treated patients, especially when the EORTC model was used (15).

Based on clinically available prognostic factors and, in particular, the data from the EORTC risk tables, the European Association of Urology (EAU) Guidelines recommend stratification of NMIBC patients into four risk groups: low, intermediate, high, and very high risk. Specific treatment recommendations have been defined for these groups (9). Low-risk patients have a low risk of disease progression and a low to moderate risk of recurrence. With the defined treatment modalities, these patients have excellent survival. Management of intermediate- and (very-)high-risk NMIBC consists of TURBT and bladder instillations with chemotherapeutics or BCG plus intensive follow-up. Despite this intensive treatment and follow-up schedule, these patients have a high risk for disease recurrence (73-84%) and a moderate risk for progression to MIBC (8.1-14%) at 5-years. On the other hand, very high-risk tumors have a very high probability of disease progression (29-54%). Equally, the rate of BCG non-responders amounts to 40% (9).

Patients with NMIBCs in the same EAU risk group can have significant heterogeneity in terms of oncological outcomes and prognosis. The failure to accurately predict outcomes of this scoring system, which is solely based on clinico-pathological features, may be partly attributed to the intrinsic heterogeneity of BC at the molecular level. The generation of large-scale, high-throughput molecular data and the development of new profiling technologies and analytical algorithms have led to molecular subtyping of the disease (16). Early molecular profiling revealed evidence for a dual-track model according to which BC develops from two distinct pathways - the papillary and the non-papillary pathway (17–19). The papillary NMIBCs develop via urothelial hyperplasia and are associated with disruption of the PI3K-AKT-mTOR (Phosphoinositide 3-kinase/Protein kinase B/Mammalian target of rapamycin) pathway and mutations in the *FGFR3* and *HRAS* genes (20, 21). The non-papillary MIBCs develop from flat dysplasia and CIS and are characterized by genetic alterations in tumor suppressor genes that regulate cell cycle and apoptosis (*TP53*, *CDKN2A*, *CCND1*, *CDKN1B*, and *RB1*) (20, 21). Although this model includes many characteristic features of BC, it does not fully address the heterogeneity of the disease (18, 19).

**Figure 1:**
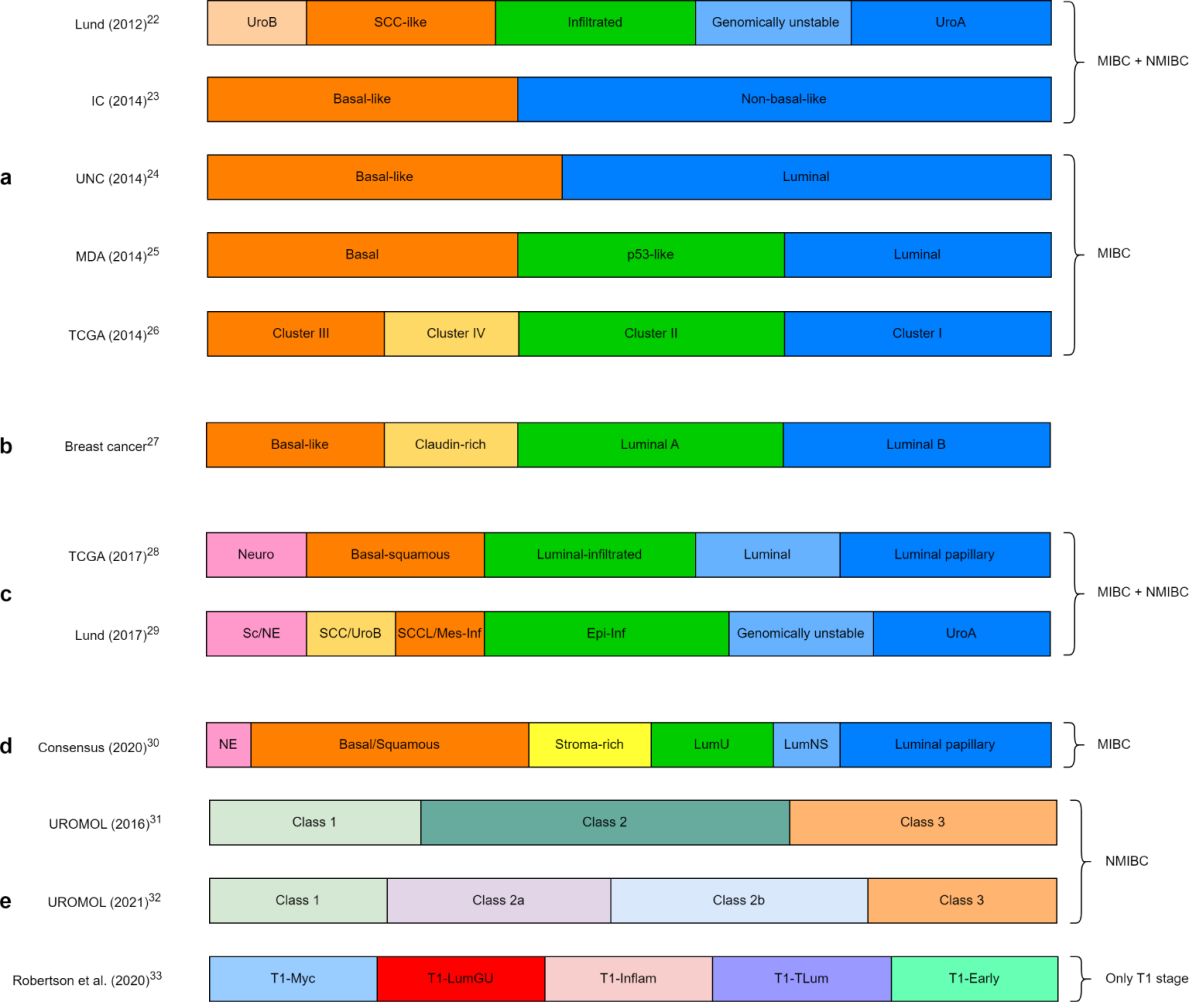
Molecular subclassification of bladder cancer by different research groups: **(A)** initial molecular classification of bladder cancer, **(B)** molecular classification of breast cancer, **(C)** recent molecular classification of MIBC, **(D)** consensus classification of MIBC, **(E)** molecular classification of only NMIBC and only T1 stage. Lund, Lund University; IC, Institute Curie; UNC, University of North Carolina; MDA, MD Anderson Cancer Center; TCGA, The Cancer Genome Atlas; UroA, Urobasal A; UroB, Urobasal B; SCC, Squamous cell carcinoma; Neuro, Neuronal; Epi-Inf, Epithelial-infiltrated; SCCL, SCC-like; Mes-Inf, Mesanchymal-infiltrated; Sc, Small-cell; NE, neuro-endocrine-like; LumU, Luminal unstable; LumNS, Luminal non-specified; LumGU, Luminal genomically unstable; Inflam, Inflammed; TLum, True luminal.

Based on gene expression, the first molecular classification of mixed samples of MIBC and NMIBC revealed five different subtypes (22). Later, two distinct subtypes, basal-like and non-basal-like, were identified again through transcriptomic analysis of MIBC with NMIBC (23). Molecular classification of only MIBC based on gene expression showed two main distinct subtypes: luminal and basal (24). Further research revealed first three different subtypes and then four subtypes, similar to those of breast cancer (**Figures 1A, B**) (25–27). The revised classifications by The Cancer Genome Atlas and Lund University showed five and six subtypes, respectively (**Figure 1C**) (28, 29). However, these subclassifications were based on DNA/RNA analyses performed mainly on retrospective cohorts of MIBC patients, with very little data from NMIBC patients. With the intense work on this subclassification, a consensus has been recently reached, which showed six different molecular subtypes in MIBC (**Figure 1D**) (30).

Comprehensive transcriptome profiling has revealed the presence of three biological subclasses in NMIBC (UROMOL Class 1, 2, and 3), thus different from that of MIBC (**Figure 1E**) (31). With the recent update of this cohort with an integrated multi-omics analysis (genomics, transcriptomics, and spatial proteomics), four subclasses (UROMOL2021 Class 1, 2a, 2b, and 3) were identified (**Figure 1E**) (32). Another recent subclassification of only the T1 stage showed five different molecular subtypes (**Figure 1E**) (33).

The current significant challenges and unmet needs are to better understand and model the disease at the molecular level, to unveil and validate new subtype-specific molecular biomarkers, and to improve the current stratification models by integrating new molecular information. Molecular data is starting to emerge in NMIBC; however, it will still take time to incorporate this data into current models. Further research is required, and then the translation of the novel findings into the clinical routine by independent randomized studies is needed for an accurate stratification of intermediate-/(very-)high-risk NMIBC to identify which patients are at the highest risk for disease progression, to predict treatment response, to identify novel targets for treatment, and to improve existing management modalities. Besides genetic alterations (mutations, copy number alterations [CNA], single nucleotide variations [SNV], insertions-deletions [indels], loss of heterozygosity [LOH], translocations, tumor mutational burden [TMB], microsatellite instability [MSI]), additional layers of information can be gained by studying control of gene activity and expression (DNA methylation, histone modification or short/long non-coding RNAs – epigenetics), RNA-RNA and RNA-protein interactions (transcriptomics), protein composition, structure and activity, and protein-protein interactions (proteomics), and unique chemical fingerprints of specific cellular processes (metabolomics-lipidomics), which might expand the current typing even further.

## 2 The hypothesis

**Figure 2:**
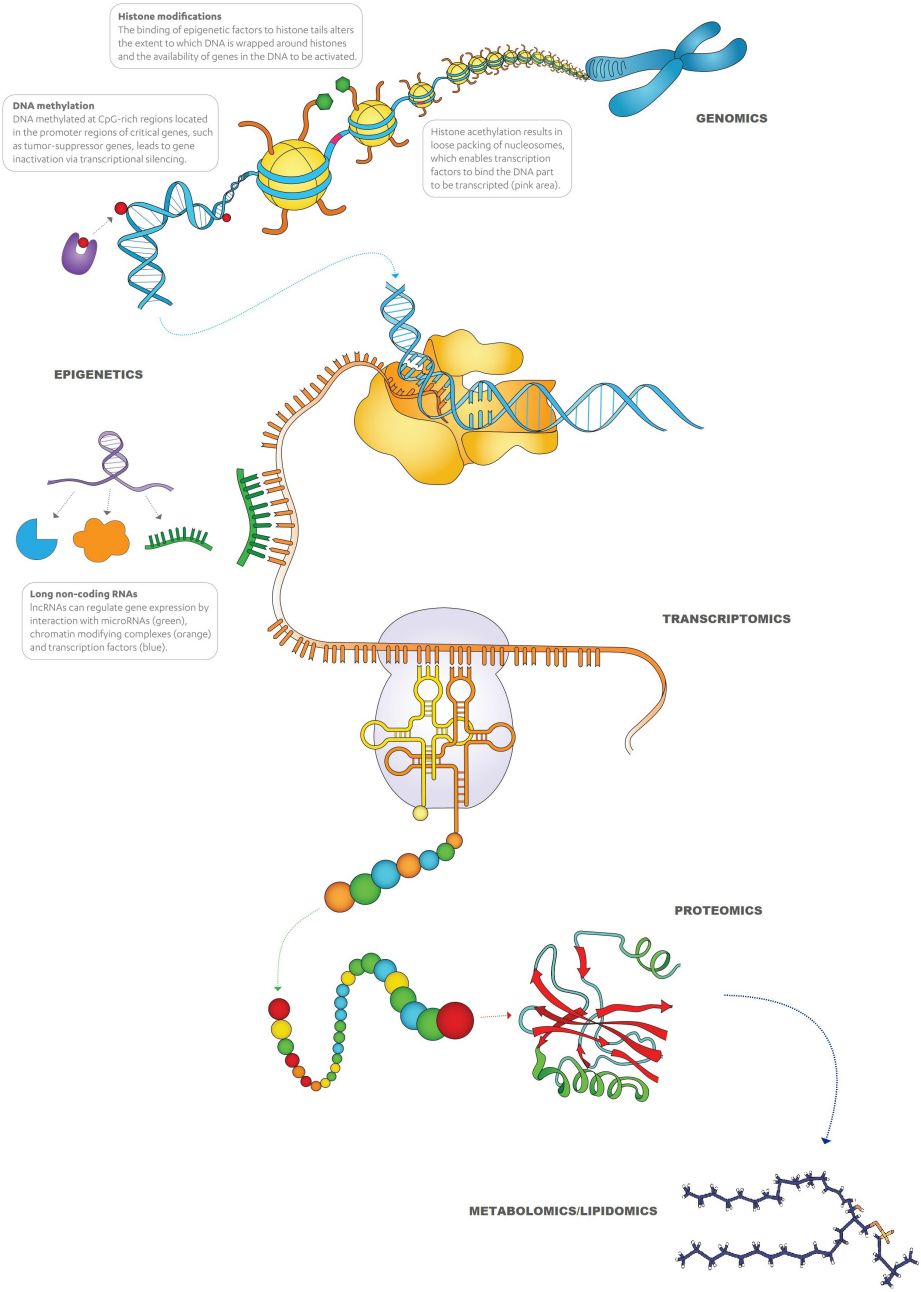
Overview of cellular processes from replication of genes to protein synthesis.

It is now well known that cell functions, such as the synthesis of peptides/proteins or other metabolites, are more complex processes than the ones explained by the central dogma of molecular biology. As **Figure 2** depicts, alterations in each step, including replication, transcription, and translation, e.g., epigenetic regulations of genes, transcriptional regulations (RNA processing), translational regulations, and post-translational modifications of proteins, and crosstalk between different processes can all be associated with the development of cancer. Apart from the ‘in-tumor’ processes, there are also other biological pathways that are driven by different cells (such as tumor microenvironment [TME], immune response, etc.) and external stimuli (carcinogens, lifestyle, radiation, infection, etc.), which play a role in the manifestation of cancer.

**Figure 3:**
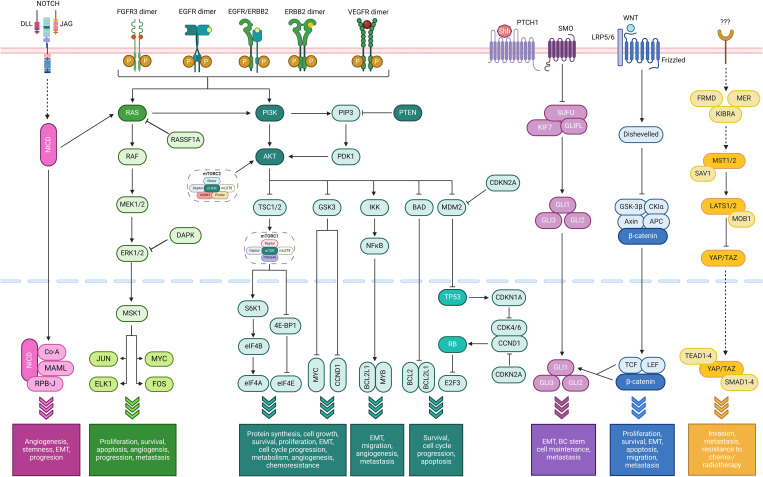
Simplified summary of oncogenic pathways playing a role in bladder cancer. From left to right: the NOTCH pathway (fuchsia), Ras/Raf/MEK/ERK pathway (bright green), the PI3K/AKT/mTOR pathway (dark green) with the TP53 and cell cycle pathways as a part of it (light green, at the very right of the pathway), the sonic hedgehog (SHH) pathway (purple), the Wnt/β-catenin pathway (dark blue), and the Hippo-YAP pathway (orange). The FGFR, VEGF, and ErbB pathways use the Ras/Raf/MEK/ERK and/or the PI3K/AKT/mTOR pathways (Created with BioRender.com).

Several pathways, commonly altered in oncogenesis and cancer progression in general, also play a significant role in BC (summarized in **Figure 3**):

### 2.1 *TP53* pathway

The tumor suppressor gene *TP53*, also known as ‘the guardian of the genome’, is the most frequently mutated gene in human tumors, and the process of tumor development is strongly related to the dysfunctions caused by *TP53* mutations. The protein coded by this gene, p53, functions primarily as a transcription factor and regulates various cell functions such as cell cycle, apoptosis, autophagy, DNA repair, and metabolism. Mutant *TP53* promotes the development and progression of BC through inhibition of apoptosis, alteration of DNA methylation patterns, activation of oncogenic pathways (e.g., PI3K/AKT/mTOR pathway), induction of multiple metabolic changes, modulation of TME with immunosuppressive changes and enhancement of metastatic potential (26, 34–37).

### 2.2 Cell cycle pathway

The cell cycle is a highly regulated pathway enabling cell growth, duplication of genetic material, and cell division. The cell cycle machinery, composed of proteins (cyclins) and their catalytic partners called cyclic-dependent kinases, drives progression from one cell-cycle phase to another. Interestingly, these proteins play a role in tumorigenesis by affecting not only tumor cells but also TME (e.g., anti-tumor immune response). Dysregulation of this pathway, through amplification or rearrangements of genes encoding D-, E-, A-, and B-cyclins (*CCND1*, *CCND2*, *CCND3*, *CCNE1*, *CCNE2*, *CCNA1*, *CCNA2*, *CCNB1*), *CDK4*, *CDK6*, *CDK2*, *CDK1*, and *CDK7*, has been documented in a vast number of cancers, including BC (26, 34, 35).

### 2.3 PI3K/AKT/mTOR pathway

This pathway is essential in regulating the cell cycle. Dysregulation of this pathway, through mutations or amplification in *PIK3CA*, loss or inactivation of *PTEN*, hyperactivation of AKT or mTOR, or inactivating mutations in *TSC1*, can lead to uncontrolled cell growth and survival, altered metabolism, increased angiogenesis and epithelial-to-mesenchymal transition (EMT), and chemoresistance (38, 39).

### 2.4 Ras/Raf/MEK/ERK pathway (also known as MAPK [mitogen-activated protein kinase] pathway)

It is a critical signal transduction cascade that transmits signals from extracellular stimuli to the nucleus, which in turn influences gene expression and impacts cell proliferation, differentiation, survival, and apoptosis. It is crucial for normal cell functions and is highly conserved evolutionarily across different organisms. It is one of the most commonly altered pathways in cancer, including BC, and its mutations or dysregulations can lead to uncontrolled cell proliferation and reduced apoptosis, which are foundational hallmarks of cancer. In BC, mutations in the components of this pathway, such as *HRAS*, lead to constitutive activation of the pathway, which promotes tumor initiation and development. This continuous activation also contributes to tumor growth and progression. It can also evade apoptosis by influencing transcription factors that regulate pro-survival genes when activated. The chronic activation of this pathway is also linked to angiogenesis, metastasis, and resistance to specific therapies, particularly those targeting upstream components like tyrosine kinase receptors (38, 40).

### 2.5 Fibroblast growth factor receptor pathway

This pathway is a critical signaling cascade involved in various cellular processes such as cell proliferation, differentiation, migration, and survival. Its activation is initiated by binding one of 22 defined fibroblast growth factors (FGF) to one of four FGFRs, which leads to the dimerization of receptor and autophosphorylation of the tyrosine kinase domain. Aberrant activation of the FGFR signaling, often due to mutations or overexpression, initiates continuous activation of several downstream pathways, including the Ras/MAPK, PI3K/AKT, and PLCγ (phospholipase C gamma). These pathways promote cellular proliferation, inhibit apoptosis, influence cell behaviors (e.g., loss of contact inhibition, anchorage-independent growth), enhance cell survival mechanisms, and alter the TME, which contributes to oncogenesis and progression of BC (41).

### 2.6 ErbB pathway

The ErbB family proteins function as cell membrane receptor tyrosine kinases, which are activated following ligand (epidermal growth factor [EGF], transforming growth factor-alpha [TGF-α], neuregulins, etc.) binding and receptor dimerization, regulate several essential cell functions such as cell proliferation, migration, differentiation, apoptosis, and motility. Their organ-specific expression plays a role in cardiac development, synaptic formation, and proliferation/differentiation of glial cells. Overexpression of *ERBB1* (encoding epidermal growth factor receptor [EGFR]=ErbB1=Her1) or *ERBB2* (encoding ErbB2=Her2) or both contributes to shorter recurrence periods and earlier disease progression in early-stage BCs (38).

### 2.7 Vascular endothelial factor pathway (also known as angiogenesis pathway)

Activation of the vascular endothelial factor receptors (VEGFRs, particularly VEGFR2) triggers angiogenesis through promoting several processes such as endothelial cell proliferation and migration, increased vascular permeability, endothelial progenitor cell mobilization, and anti-apoptotic effects, which in turn end up with tumor growth, progression, and metastasis (38, 40).

### 2.8 NOTCH pathway

It is a highly conserved cell signaling system present in most animals and plays a major role in neurogenesis and regulation of embryonic development. This pathway has a dual function in BC, where it suppresses proliferation by upregulating dual-specificity phosphatases when activated and leads to tumorigenesis by *ERK1/2* phosphorylation when inactivated. Inactivation of this pathway, through loss-of-function mutations in *NOTCH1* and *NOTCH2*, loss of *HES1* expression, and overexpression of *NOTCH3* and *JAGGED2*, contributes to tumor angiogenesis, stemness, EMT, and cancer progression (42).

### 2.9 Sonic hedgehog pathway

It plays a critical role in organ development, acting as a morphogen involved in patterning many systems, such as several parts of the central nervous system, lungs, teeth, limbs, and digits, and regulating the proliferation and differentiation of adult stem cells. Dysregulation of this pathway by means of mutations in genes encoding its components (e.g., *PTCH1*, *SMO*, *GLI*), epigenetic modifications in the promoter regions of the pathway genes, overexpression of pathway components (SHH ligands, transcription factors), and crosstalk with other signaling pathways, drives key processes such as EMT, BC stem cell maintenance, and lymph node or distant metastasis (43).

### 2.10 Wnt/β-catenin pathway

This pathway is highly conserved across multiple species and critical to both embryological development and adult tissue homeostasis regeneration. Aberrant activation of this pathway, often through mutations or epigenetic alterations in the pathway components (e.g., *APC*, *CTNNB1*, *PTEN*, Wnt ligands or antagonists), leads to nuclear accumulation of β-catenin and subsequent transcriptional activation of Wnt target genes (e.g., *MYC* [*c-Myc*], *CCND1*), which in turn contribute to uncontrolled proliferation, evasion of apoptosis, promoting EMT, and enhanced metastatic potential (44).

### 2.11 Hippo-YAP pathway (also known as MST/WW45/LATS pathway)

It is involved in cell growth, apoptosis, homeostasis, and controlling organ size during embryonic development. Dysregulation of this pathway, through, e.g., overexpression of transcription factor YAP (yes-associated protein) or transcription coactivator TAZ (PDZ-binding motif) or decreased expression of *MST1/2* and *LATS1*, plays a role in invasion, metastasis, and resistance to the cytotoxic effects of chemotherapy (especially cisplatin) and radiotherapy (26, 35, 40).

### 2.12 Histone modification (chromatin regulatory) pathway

Histone modifications such as acetylation, methylation, phosphorylation, ubiquitination, SUMOylation, and ADP-ribosylation regulate chromatin structure and gene expression. Dysregulation of specific histone-modifying enzymes such as histone acetyltransferases, histone deacetylases (HDACs), histone demethylases or their catalytic subunits (e.g., EZH2), through overexpression and loss-of-function mutations in the encoding genes (*KDM6A*, *HDAC1*, *HDAC2*, *HDAC3*, *EZH2*, *MLL2* [*KMT2D*], *SETD2*), plays role in cancer initiation, increased genomic instability and aggressiveness, stemness, progression, and metastasis (26, 35, 38).

### 2.13 SWI/SNF (SWItch/Sucrose Non-Fermentable) complex

This complex is a subfamily of ATP-dependent chromatin remodeling proteins and regulates transcription of specific genes by altering the chromatin structure and functions as a tumor suppressor in cancers. Mutations in the genes encoding the subunits of the SWI/SNF complex such as *ARID1A* (the most frequent), *ARID1B*, *SMARCA4*, *SMARCA2*, *SMARCC2*, *SMARCC1*, and *PBRM1*, promote several key hallmarks of cancer such as cell proliferation and survival, invasion, stemness, and interactions with the other oncogenic pathways, which in turn increases the aggressiveness of BC (26, 40).

Apart from the abovementioned ones, there are also other pathways involved in tumorigenesis and progression of BC, which play a role in cancer cell metabolism and cancer stem cells such as IL6/IL6R/STAT3, COX2/PGE2/SOX2, ALDH1A1/TUBB3, ARRB/ALDH/CD44 pathways (40). External carcinogenic stimuli such as cigarette smoke and occupational exposure cause BC development through several mechanisms, including formation of DNA abducts and oxidative DNA damage (e.g., 8-oxodeoxyguanosine), accumulation of somatic mutations, aberrant DNA methylation (hypermethylation of tumor suppressor genes, hypomethylation of oncogenes), histone modifications (acetylation, methylation, other post-translational modifications), microRNA dysregulation, platelet-activating factor (PAF) accumulation, activation of multiple pathways (e.g., *STAT3* and *ERK1/2* by nicotine), and affecting EMT process (45, 46). Even though the effect of smoking on disease recurrence has been widely studied, it is less investigated for disease progression, and the results have been ambiguous (47). Very few studies could find an association between smoking and progression. In contrast, the others have failed to do so, probably due to the limited power of studies because of a relatively small number of patients and/or events and progression being not the primary endpoint of those studies. A recent systematic review and meta-analysis has found that the risk of progression was not increased for smokers vs. never-smokers, while ever-smokers had a compromised progression-free survival both for all patients and subgroups of high-risk and BCG-treated patients (48). On the other hand, several studies have shown that cigarette smoke induced the initiation and progression of BC and mediated the EMT and *ERK1/2* pathway (49). A very recent study using single-cell and multi*–omics* analyses identified 33 tobacco carcinogens-related genes and constructed a prognostic score that showed high-risk patients had significantly worse overall survival. This study also highlighted that cancer-associated fibroblasts mediated the crosstalk between EMT and immune evasion, which in turn played a role in disease progression (50). A serum metabolic profiling study identified 40 metabolites, including an increased abundance of amino acids (tyrosine, phenylalanine, proline, serine, valine, isoleucine, glycine, and asparagine) and taurine, in smoker BC patients. An integromic analysis of differential metabolomic gene signature and transcriptomics data revealed an intersection of 17 genes (catechol-O-methyltransferase, iodotyrosine deiodinase, and tubulin tyrosine ligase being the most important ones) that showed a significant correlation with the survival of smokers with BC (51).

Even though our current knowledge about how these oncogenic pathways is dysregulated has increased enormously with recent studies, there are still unknowns. More importantly, revealing these pathways individually is not enough, as the crosstalks between these pathways make the whole process much more complicated. Understanding the missing parts and the relationships of these pathways with each other can facilitate the development of novel diagnostic and therapeutic strategies. Moreover, our knowledge is fragmented as most studies focus on only one specific oncogenic pathway. We need to look broader to understand the whole picture. Current technology allows for such multiplatform analyses.

There is an unmet need to develop a more accurate classification system in NMIBC. A better patient stratification for specific follow-up regimens and selected treatments, based on prediction of disease prognosis and response to treatment, is needed. This will require the assessment of multiple biological parameters. Based on these assumptions, by employing a multi-layered *–omics* (namely, genomics, epigenetics, transcriptomics, proteomics, lipidomics, metabolomics) and immunohistopathological approach, we hypothesize to demonstrate molecular heterogeneity of intermediate- and (very-)high-risk NMIBC and to stratify the patients better.

Overestimation of the risk of disease recurrence and progression in high-risk patients by the EORTC and the CUETO risk calculators and the fact that some researchers have failed to validate these calculators externally can be explained by the inherent disease heterogeneity. The molecular characterization of MIBC has already shown several subgroups with clearly different characteristics (21–29). The same has recently been proved to be true also for NMIBC (30–32).

These facts support our hypothesis that a combinatorial multi*–omics* approach will be more powerful for detecting and explaining existing disease heterogeneity. Each *–omics* layer provides unique and different but limited information; however, only combining several *–omics* with high-performance data linkage using powerful bioinformatics will unravel a better, more detailed, and multi-dimensional characterization of disease mechanism and (novel) treatment targets. Meanwhile, the transfer of the results of multi*–omics* into immunohistopathology will allow us to use the new data in routine daily practice, even without the need for sophisticated infrastructure.

For this reason, we propose to perform a comprehensive multilayer assessment of the genome, epigenome, transcriptome, proteome, lipidome, metabolome, and immunohistopathological characteristics that represent the broad spectrum of NMIBC phenotypes. This has the potential to improve the prediction of recurrence and progression and to develop an algorithm (based on clinical, pathological, and molecular features) to adequately stratify patients and guide therapeutic interventions in a personalized manner. The lack of predictive value of the currently used risk calculators results in under- or over-treatment of patients, which ultimately leads to poor quality of life, observed high BC fatality rates, and increased treatment costs (11, 52, 53). Moreover, a comprehensive depiction of the mechanism(s) of non-responsiveness to intravesical BCG treatment of NMIBC will reveal unique molecular pathways that can further guide drug development in the future by identifying novel therapeutic targets.

## 3 Evaluation of the hypothesis

Recent technological improvements in molecular biology have tremendously increased our knowledge of the biological character of cancer, including BC. The data from different *–omics* studies help us unravel the molecular complexity of BC more efficiently.

### 3.1 Genomics

Increasing evidence suggests that genetic mutations (germline or somatic) significantly influence the incidence of BC (54, 55). For this reason, most research has focused on detecting genomic alterations. The most recent studies performed on a large number of patients with NMIBC showed that the most frequently altered genes are *TERT* promoter (73%), *FGFR3* (49%-34%), *KDM6A* (38%-18%), *PIK3CA* (26%-25%), *STAG2* (23%-10%), *ARID1A* (21%-5%), *TP53* (21%-9%), *FAT1* (15%-11%), *KMT2D* (24%-12%), and *KMT2C* (11%-10%) (the first and second percentages come from Ref. #56 and #32, respectively). RTK/PI3K, TP53/cell cycle, chromatin modification, and DNA damage repair (DDR) are frequently altered pathways (32, 56). The most frequent CNAs in BC involve *CDKN2A*, *TP53*, *FGFR3*, *HRAS*, *ERBB2*, *TSC1*, *RB1*, *PTEN*, *CCND1*, *MDM2*, and *E2F3* (56–58). 

TMB is defined as the total number of somatic missense mutations per megabase (Mb) of the tumor’s genomic DNA. It serves as a biomarker for predicting response to immunotherapy, with higher TMB often correlating with better prognosis and increased sensitivity to immune checkpoint inhibitors. In BC, a threshold of 10 mutations/Mb is commonly used to distinguish high from low TMB. Key genes involved in BC with high TMB include *TP53*, *KMT2D*, *KDM6A*, *ARID1A*, *KMT2C*, *PIK3CA*, *FAT4*, *EP300*, and *RB1*. Additionally, some of the MMR (*MSH2*, *MSH6*), DDR (*ATM*, *BRCA2*, *POLQ*, *CDK12*, *ATR*, *BRIP1*), and polymerase-encoding genes (*POLE*, *POLD1*) are frequently altered in TMB high tumors (59, 60). In the 100,000 Genome Project, the UC of the bladder was found to have median mutations of 7.2/Mb, and 11.9% of the cases had a high TMB, defined as >20 mutations/Mb (61). 

Resulting from genomic hypermutability, variations in the length of repetitive sequences (microsatellites) in the entire genomic structure are known as MSI. In tumor cells with MSI, DNA mismatches in microsatellites cannot be repaired due to a deficient MMR machinery, which results in the accumulation of mutations in tumor suppressor genes and/or oncogenes (62). Recent studies have shown that MSI can be used as a predictor of response to immune checkpoint inhibition in various solid organ tumors as well as BC (62). Microsatellite analysis (MSA) can be used to identify both initial or recurrent tumors, with a better sensitivity and specificity than urine cytology, where low-grade and low-stage disease can be detected as accurately as high-grade and high-stage disease (62). LOH is identified by comparing the DNA isolated from tumor tissue to normal DNA, generally isolated from blood, with the MSA. LOH at 9p, 17p, 9q, 8p, 13q, 11p, and 4p have been shown to have prognostic value in NIBC (62).

### 3.2 Epigenetics

Although genetic mutations are mainly investigated, epigenetics represents more prevalent DNA alterations that can lead to the development and progression of cancer. Epigenetic alterations can be defined as stable molecular changes of the phenotype of a cell that are inheritable during somatic cell divisions (and sometimes germ line transmissions) but do not involve changes in the DNA sequence itself. The major epigenetic phenomena in cancer cells are mediated by several molecular mechanisms comprising DNA hypermethylation, histone modifications, nucleosome remodeling, and RNA-associated silencing (**Figure 3**). 

The most studied epigenetic mechanism of these is DNA hypermethylation that occurs in CpG islands (a cytosine that precedes guanine in a CpG dinucleotide) in promoter regions. Besides the methylated genes common to various cancer types (*GSTP1*, *CDKN2A*, *RB1*, *MLH1*, *APC*, *PTEN*, *DAPK1*, *MSH6*, *MGMT*, *RASSF1A*, *TIMP3*, *BRCA1*, *CDH1*, *VHL*, *CDKN2B*, *FHIT*, *TWIST1*, *ONECUT2*, *WIF1*, *HIC1*, *PRAC1*, *SFRP5*, *RUNX3*, *SOCS1*, etc.) and more specific to BC (*ZNF154*, *HOXA9*, *POU4F2*, *EOMES*, *ACOT11*, *PCDHGA12*, *CA3*, *PTGDR*, *TBX4*, *FGFR3*, *PMF1*, *PCDH8*, *PCDH17*, *GDF15*, *KISS1*, *ISL1*, *ALDH1A3*, *TBX3*, etc.), new candidate genes will be found as further studies are performed, which can be used for screening, diagnostic and prognostic purposes (63–65).

### 3.3 Transcriptomics

The majority of the molecular research performed on BC depends on transcriptomic analyses. With gene expression studies by using messenger (m) RNA, several molecular subclassifications of BC have been performed. First, binary subtyping, namely luminal and basal, has been proposed (22–24). Later, with further research, up to six subtypes have been identified by different research groups (25–29). With an effort to mitigate the differences and inconsistencies among these molecular subtypings, a consensus subclassification has been performed, which showed six different molecular subtypes: luminal papillary, luminal non-specified, luminal unstable, stroma-rich, basal/squamous, and neuro-endocrine-like (30). Transcriptomic profiling of only NMIBC identified three molecular subtypes (UROMOL2016 Class 1, 2, and 3), which has been recently updated with employing multi*-omics* and revealed four different subclasses (UROMOL2021 Class 1, 2a, 2b, and 3) (31, 32). 

Apart from molecular subtyping studies, numerous research focused on the differential expression of specific genes and their effect on BC formation, progression to muscle-invasive or metastatic disease, prognosis, and response to chemotherapy or immunotherapy. Recent research on micro (mi) RNAs revealed their roles in stratifying patients, detecting disease progression, and predicting clinical outcomes in BC, which have the potential to be used as promising biomarkers (66, 67). An increasing number of recent studies on long non-coding (lnc) RNAs showed their roles in proliferation, differentiation, migration, invasion, apoptosis, and metabolism (e.g., glycolysis) of tumor cells, resistance to cisplatin, stemness, and EMT (68, 69). Additionally, circular (circ) RNAs, another type of small non-coding RNAs, have emerging oncogenic and anti-oncogenic functions, particularly regulating migration, invasion, and drug resistance, in BC (70, 71).

### 3.4 Proteomics

Proteomics can be broadly classified into discovery and targeted proteomics, which are highly complementary to each other. Discovery proteomics is predominantly conducted using mass spectrometry (MS)-based technologies, which allow comprehensive analysis of proteins and post-translational modifications without the requirement of generating target-specific antibodies. However, it still has several technical challenges, and newer methods are being developed to make it more efficient by increasing its dynamic range of peptide sampling and resolution (72, 73). Liquid chromatography-coupled tandem mass spectrometry (LC-MS/MS) is the gold standard for current proteomics research; however, it cannot identify and quantify specific proteins in complex mixtures with a similar scale and sensitivity to that of next-generation DNA sequencing. The proteomic data can be used for several purposes, such as screening (detection of new or recurrent disease), patient stratification, prediction of treatment response, and identification of novel drugs/drug targets (74). 

A recent systematic review has identified the top ten enriched pathways of proteomic biomarkers for BC, namely the immune system, innate immune system, complement cascade, integrin beta 3 cell surface interactions, mesenchymal-to-epithelial transition, EMT, FGFR signaling, c-Myb transcription factor network, endogenous TLR signaling, and Trk receptor signaling mediated by the MAPK pathway (75). Strogglios et al. reported the first proteomic classification of 98 NMIBC samples based on an unbiased comprehensive LC-MS/MS approach, in which three NMIBC proteomic subtypes (NPS) were identified: NPS1 (mostly high stage/grade/risk samples) was the smallest group (17.3%) and overexpressed proteins reflective of an immune/inflammatory phenotype, involved in cell proliferation, unfolded protein response, and DNA damage response. While NPS2 (mixed stage/grade/risk composition) presented with an infiltrated/mesenchymal profile, NPS3 had differentiated/luminal phenotype, in line with its pathological composition (mostly low stage/grade/risk samples) (76). Based on The Cancer Genome Atlas (TCGA) dataset, the immune-related prognostic signature (IRPS) was constructed with seven immune-related genes (*STAT3*, *TGFB1*, *CTSG*, *NFKB1*, *SNRPD2*, *PDCD1*, and *TAP1*). It was related to poor five-year overall and disease-free survival (77). Dressler et al. have analyzed 242 tumor samples from different stages and identified five proteomic subtypes: PAULA (Proteomic Analysis of the Urothelial cancer LAndscape) 1 was a low-risk cluster with the highest number of samples and the longest survival, where PAULA IIa/IIb/IIc were the intermediate-risk clusters, and PAULA III was the high-risk cluster with the shortest survival (78). While some studies present proteins specifically for disease stage (e.g., coded by *PRDX1*, *UMP/CMPK*, *GSTM1*, *PGAM1*, *PRDX6*, *PSME1*, *HSPB-1*, *ANXA1*, and *CAPG* for NMIBC; *BLVRB*, *PRDX2*, and *HPGD* for MIBC), recent studies reveal novel potential biomarkers for BC in general such as *RET*, *PVRL4*, *AREG*, *FGFBP1*, *WFDC2*, *ESM-1*, *SPR*, *AK1*, *CD2AP*, *ADGFR1*, *GMPS*, and *C8A* (79–81).

### 3.5 Lipidomics & metabolomics

Even though the exact mechanism is not still clearly unraveled, it has been known for a long time that most cancer cells produce their energy predominantly through anaerobic glycolysis followed by lactic acid fermentation, which is known as the Warburg effect, instead of the usual citric acid cycle and oxidative phosphorylation. Recent research has proven that both cancer development and metastatic disease progression are characterized by a unique reprogramming of cellular energy, glucose, and lipid metabolisms, which is required for the maintenance of rapid proliferation of cancer cells (82–85). Moreover, the deregulation of cellular metabolism is now considered one of the hallmarks of cancer (86). 

Recent studies have shown that a variety of characteristic metabolic changes, including increased glucose utilization for glycolysis and *de novo* fat synthesis, elevated sorbitol pathway intermediates, oxidative metabolism imbalance, glutamine consumption, altered metabolism of membrane lipids, and differential derivation of nucleic acid components pyrimidine and purine, are observed in NMIBC and MIBC (87–89). Piyarathna et al. have reported a progressive decrease in the levels of phosphatidylserine, phosphatidylethanolamines, and phosphocholines, whereas an increase in the levels of diacylglycerols with increasing tumor stage in UC. The levels of diacylglycerols and lyso-phosphatidylethanolamines were significantly elevated in tumors with lymphovascular invasion and lymph node metastasis, respectively (90). Comparative lipidomic profiling of two isogenic human T24 BC cell lines showed reprogrammed lipid metabolism was associated with cisplatin resistance (91). These findings encourage further research to identify various potential biomarkers for non-invasive diagnosis and also for prediction of recurrence and progression in BC.

While these ‘bulk’ profiling methods have offered invaluable insights into the key biological events and the molecular characteristics of mechanistic pathways involved in BC, they lack the ability to show intratumoral heterogeneity, as tissue specimens are processed as a whole and the data originating from different components of the tumor (e.g., tumor cells, immune cells, endothelium, connective and/or muscle tissue cells) cannot be recognized separately. At this point, state-of-the-art technologies such as single-cell/-nucleus sequencing and spatial *–omics* help fill the existing gaps and increase our knowledge. Very recent research using single-cell and/or spatial transcriptomics, proteomics, and metabolomics has substantially augmented our pre-existing corpus of knowledge by improving our perception of the molecular basis of the intratumoral heterogeneity and tumor cell-TME interaction in BC (92–95).

Even though it has been less than a decade since these two technologies have been commercially available, the number of techniques has increased tremendously, and every single new method tries to compensate for the disadvantages or hurdles of the existing approaches (96). However, there are still some aspects that need to be improved, such as detection efficiency, transcriptome-wide profiling, spatial resolution, sequenced tissue section area, cost, and tissue compatibility/usability. Most of these methods are applicable only to fresh-frozen (FF) tissues, while very few techniques have been implemented in formalin-fixed paraffin-embedded (FFPE) tissues, such as deterministic barcoding in tissue sequencing (DBiT-seq), CellScape (Canopy, Biosciences, St. Louis, MO, USA), and Visium Spatial and Xenium *In Situ* (10x Genomics, Pleasanton, CA, USA) (97). While FF tissues are disadvantageous as they are inappropriate for prolonged storage, prone to deformation over time, and gene diffusion during tissue permeabilization, the RNA in FFPE samples is of inadequate quality.

On the other hand, whether it is spatial or not, sequencing only one of the abovementioned *–omics* provides information from a single aspect of the tumor. It would be more promising if altered (mutated, under-/overexpressed) genes, control mechanism(s) and mediator(s) of their (in)activation, expressed and (possibly) modified proteins and their interaction between each other, altered metabolites of the cellular functions, and histopathological anatomy of the cancer tissue could be viewed simultaneously, preferably from the same slide. This holistic approach would extensively deepen our knowledge of cancer pathophysiology. By providing invaluable information from different aspects of the tumor, the (spatial) multi*–omics* technology provides a comprehensive understanding of the functions and regulations of driver genes, expressed proteins, and metabolites (mid-/end-products of different pathways) for cancer initiation and progression. When it is implemented for NMIBC, this multi-layered large-scale data will help to improve molecular and clinical subtyping, delineate tumor cell behavior, predict tumor response to treatments, find novel druggable targets, detect tumor development, recurrence, or progression with more efficient liquid biopsies, and to support clinical decision-making.

For testing the combinatorial approach of multi*–omics* in NMIBC, biosamples (urine, blood, and BC tissue) should be collected prospectively from all patients who are planning to undergo TURBT, as some of the *–omics* studies mentioned here can only be performed on FF biosamples. According to predefined standard operating procedures for each *–omics* study, biosamples should be collected and stored immediately at -80°C till analyses are performed. Multi*–omics* data have value only when combined with long-term follow-up data, which should be collected simultaneously from the same patient cohort. Furthermore, longitudinal molecular profiling of cancer tissue, urine, or blood during patient follow-up will reveal the disease’s evolution when there is a recurrence or progression. At this point, accurately identifying novel biomarkers showing the presence of recurrence and/or progression can potentially decrease the number of cystoscopies and/or imaging performed during the follow-up.

It is obvious that an enormous set of data points will be generated with the combination of the abovementioned multi*–omics* approach. Here, researchers face another big challenge: Previous approaches, such as the Pearson/Spearman correlation and the Kaplan-Meier method, could only perform pairwise data integration and were insufficient to process multi-layered big data. Recent advancements in mathematical methods, such as matrix deconvolution, network approaches, and machine learning, have significantly enhanced multi*–omics* data integration. Bioinformaticians have developed new, specialized bioinformatic pipelines necessary for collecting, processing, and manipulating *–omics* data to integrate their associations with clinico-pathological features. Key steps for data collection, preparation, representation, and clinical use in a multi*–omics* approach can be summarized as follows (98):

- Data collection: Raw data, consistent in experimental conditions and data format, are collected from different *–omics* platforms and then converted into quantitative data.
- Data cleaning and quality assessment: Missing values and outliers are identified, and data quality control metrics are used to ensure data reliability before downstream analyses.
- Normalization: Data normalization methods are used for different *–omics* layers to mitigate biases and to ensure comparability and compatibility.
- Feature selection: Genomic and epigenetic alterations, differential gene expression, and other proteomic/metabolomic/lipidomic abnormalities are identified, and relevant features are selected based on biological and/or statistical significance. Filter methods (e.g., correlation analysis), wrapper methods (e.g., recursive feature elimination), and embedded methods (e.g., LASSO regression) are commonly used feature selection approaches. Dimensionality reduction methods such as principal component analysis (PCA), t-distributed stochastic neighbor embedding (t-SNE), uniform manifold approximation and projection (UMAP) are used to mitigate computational complexity and to facilitate appropriateness of data input for different analysis tools.
- Data integration: Frequently used computational methods and their available tools (in brackets) include PCA [Scikit-learn], canonical component analysis (CCA) [MixOmics], independent component analysis (ICA) [FastICA], non-negative matrix factorization (NMF) [NIMFA], Tensor factorization [TensorFlow], multiple kernel learning (MKL) [MKLpy], regularized regression models [GLMNET], ensemble methods [DIABLO], network-based integration [OmicsNet], and deep learning models [Keras, TensorFlow].
- Annotation: The integrated data are annotated with relevant biological and functional information such as gene ontology terms, gene regulatory network, metabolic pathways, and signaling pathways. There are various publicly available pathway databases for different purposes, such as KEGG (Kyoto Encyclopedia of Genes and Genomes), WikiPathways, Reactome, PANTHER (Protein ANalysis THrough Evolutionary Relationships), TRANSFAC (TRANScription FACtor database), Pathway commons, etc.
- Data fusion: The integrated data are fused into a cohesive representation so that the information from different *–omics* layers is combined.
- Clustering & subtyping: Unsupervised or supervised clustering techniques are employed to identify clusters or subtypes within the integrated data, which in turn provide insights into tumor heterogeneity.
- Machine learning modeling: Unsupervised (k-means clustering, hierarchical clustering, Gaussian mixture models) or supervised (support vector machines, random forests, neural networks, logistic regression, decision trees) learning techniques are used to group samples based on similar *–omics* profiles and predict outcomes or uncover patterns within the multi*–omics* data.
- Visualization: Several data visualization tools, such as scatterplots, heatmaps, network plots, circos plots, hexbins, are used to understand the variability and subpopulations within datasets. Interactive visualization tools enable researchers to explore and analyze multi*–omics* data interactively.
- Interpretation: Interpreting the findings in the context of biological knowledge, pathways, and functional relevance helps not only understand the biological significance of observed patterns or specific subpopulations but also find novel information and decipher biological heterogeneity.
- Validation: The validity, robustness, reproducibility, and generalizability of the findings from the integrated data should be ensured using rigorous validation methods, cross-validation techniques, and independent datasets.

Various multi*–omics* tools such as iCluster, PARADIGM (PAthway Recognition Algorithm using Data Integration on Genomic Models), MetScape 2, BCC (Bayesian Consensus Clustering), SNF (Similarity Network Fusion), LRAcluster (Low Rank Approximation based multi*–omics* data clustering), PaintOmics 3, iOmicsPASS, SALMON (Survival Analysis Learning with Multi-Omics Neural Networks), NEMO (NEighborhood based Multi-Omics clustering), MONET (Multi Omic clustering by Non-Exhaustive Types), PIntMF (Penalized Integrative Matrix Factorization), MergeOmics 2.0, OmicsAnalyst, Arena3D, NeDRex, OmicsNet 2.0, DriverDBv4, are currently being used to integrate multi*–omics* data (99–102). However, these tools rely on different mathematical theories and computational approaches from each other and can support different data types. Therefore, to reach the defined goal with the multi*–omics* data, researchers should select appropriate multi*–omics* tool(s) according to their data type. Moreover, they are not very user-friendly and require advanced skills and experience in R, Python, or MATLAB. As the data are different from bulk multi*–omics* data, specific computational methods have been developed for the integration of data originating from single-cell multi*–omics* platforms such as MOFA+ (Multi-Omics Factor Analysis), scAI (single-cell Aggregation and Inference), scMVAE (single-cell Multimodal Variational Autoencoder), DCCA (Deep Cross-omics Cycle Attention), citeFUSE, and Seurat v4 (103, 104). With the recent developments in artificial intelligence (AI), various AI-based computational tools for multi*–omics* data integration have been developed for different purposes such as molecular subtyping, prediction of drug response, survival prediction, patient clustering (e.g., OmiEmbed, MetaCancer, DeepDRK, PathME, DeFusion, AKLIMATE, PRODeepSyn, etc.) (105). However, there is still ample room for further studies to develop newer computational methods for better, more proper, and robust multi*–omics* data integration, enabling systematic assessment of multi-layered findings.

**Figure 4:**
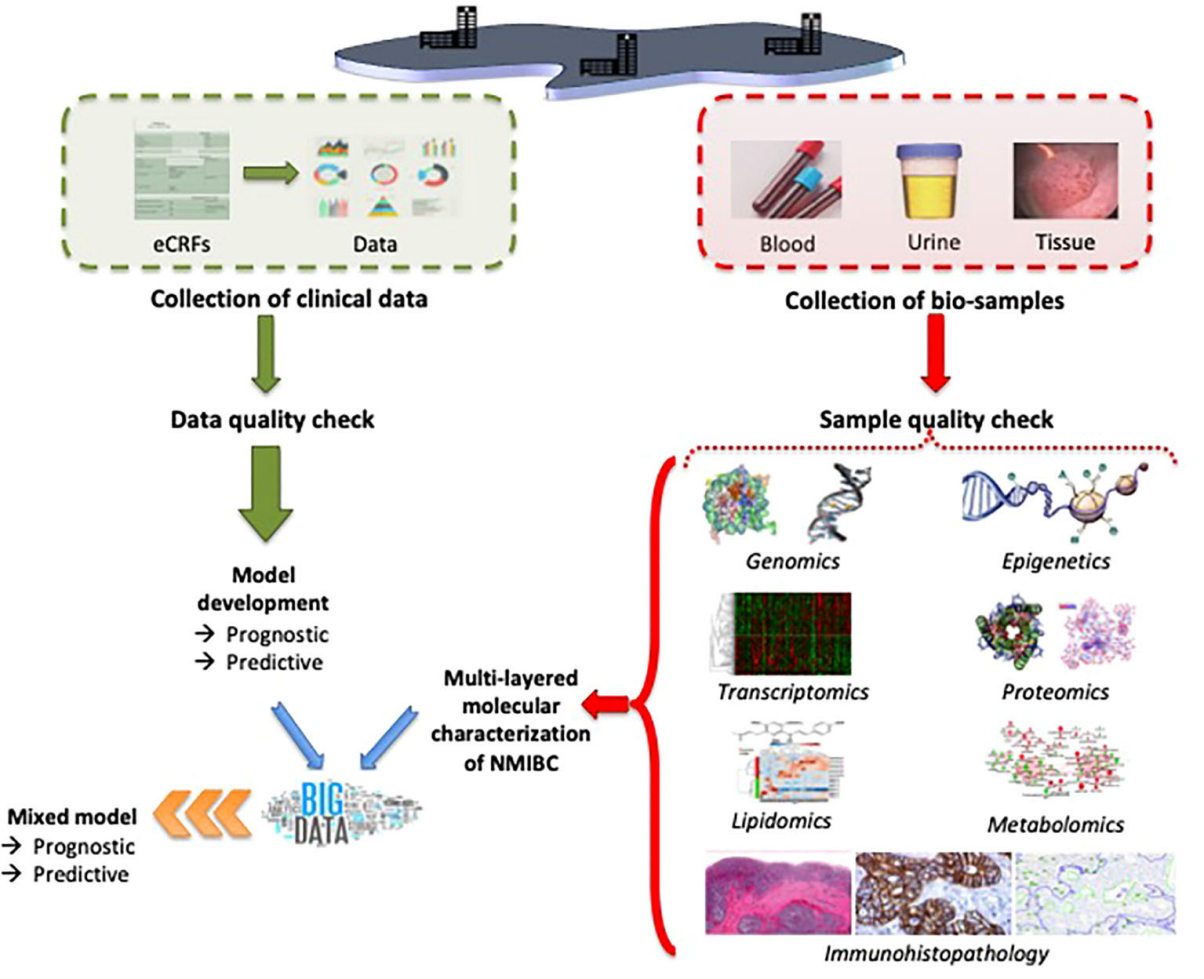
Possible setup of the experimental method for the characterization of intermediate- and high-risk NMIBC by using multi*–omics*.

When the multi*–omics* pipelines are used for NMIBC, the integrated multi-layered data would allow to identify significant associations of genome, epigenome, transcriptome, proteome, lipidome, metabolome, and immunohistopathological profiles to improve stratification of intermediate- and (very-)high-risk patients, and to develop classifiers for predicting disease outcomes and response to treatment (e.g., discriminating high-risk tumor profiles that have a higher potential to progress to MIBC and not to respond to intravesical BCG treatment) (**Figure 4**). The clinical performance of these classifiers should be tested (specificity, selectivity) and compared to the currently used criteria (EAU risk groups and risk stratification according to Gontero et al.) (12, 106). The analytical assay should be validated regarding repeatability, intermediate precision, and reproducibility.

We know that testing this hypothesis in the abovementioned setting will have some limitations. As the number of parameters that will come out from each *–omics* is not precise in the beginning, it may not be easy to determine the number of patients required for the training and validation cohorts. This would affect the power analysis and create a risk of ‘over-fitting’; however, this risk can be eliminated using the Bonferroni correction and more sophisticated computational methods.

## 4 Consequences of the hypothesis and discussion

Intensive work is currently being done in the field of BC markers with the goal of characterizing BC earlier, both at the initial diagnosis and at recurrence and/or progression. Although various *–omics* biomarkers have been identified for disease recurrence and progression up to now, the study of BC biomarkers is still in its developmental state.

The current major challenge and unmet need are twofold. First, there is a need for real-life contemporary data on NMIBC patients, which can be provided by setting up a multicentric organization that merges the results of already existing datasets. The second unmet need is the development of a more accurate scoring system, which takes into account the treatment they received after TURBT, to enable better stratification of the intermediate- and (very-)high-risk NMIBC patients for treatment selection and further follow-up scheduling, also based on a better prediction of response to treatment.

**Figure 5:**
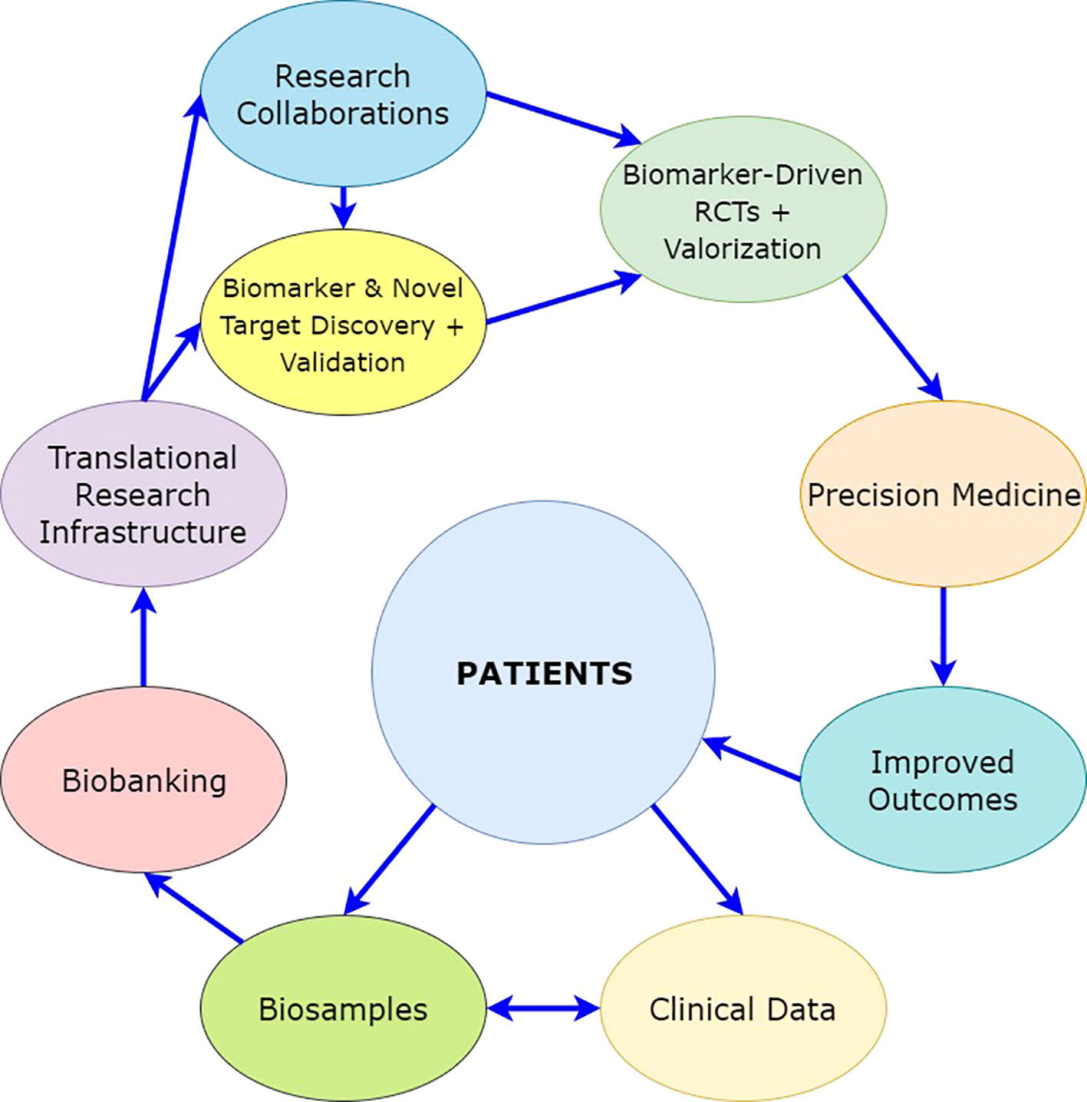
The interaction of the elements of the hypothesized risk stratification.

With this hypothesis, the aim is to define the tumor at the molecular level using high-resolution multi-layered *–omics* profiling and to use the molecular and clinical data to guide therapeutic intervention at a personalized level for NMIBC. A comprehensive depiction of the mechanism(s) of non-responsiveness to intravesical BCG treatment of NMIBC will reveal unique molecular pathways that can further guide drug development in the future by providing novel therapeutic targets (**Figure 5**).

**Figure 6:**
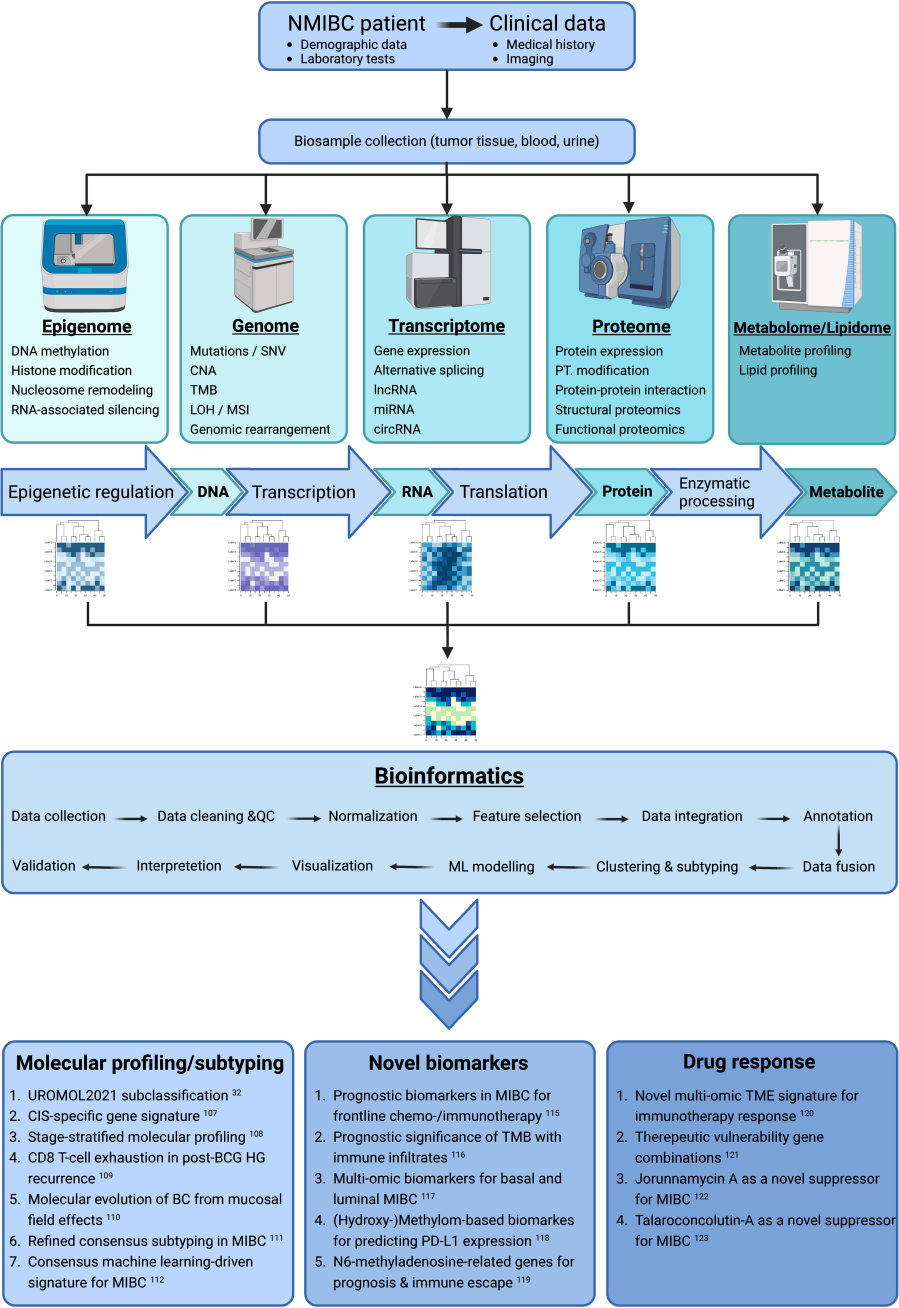
Simplified schema for multi*–omics* integration strategies in BC with examples of recently published papers for the use of findings (Created with BioRender.com). BC, Bladder cancer; BCG, Bacillus Calmette-Guérin; circRNA, Circular RNA; CIS, Carcinoma *in situ*; CNA, copy number alteration; HG, High grade; LOH, Loss of heterozygosity; lncRNA, Long non-coding RNA; MIBC, Muscle-invasive bladder cancer; miRNA, Micro RNA; ML, Machine learning; MSI, Microsatellite instability; NMIBC, Non-muscle-invasive bladder cancer; PD-L1, Programmed death ligand 1; PT, Posttranslational; QC, Quality control; SNV, Single nucleotide variation; TMB, Tumor mutational burden; TME, Tumor microenvironment.

A rapidly increasing number of research has been recently published in which disease heterogeneity, patient stratification, predictive and/or prognostic biomarkers, and cancer drug response were targeted to be improved using the bi-/tri-/multi–omics approach. Some of these studies with important molecular or clinical implications are summarized here (**Figure 6**). Among these critical researches, the most important can be emphasized as the tri–omics (genomics, transcriptomics, proteomics) approach improving the existing UROMOL2016 subclassification of NMIBC by identifying four different prognostic molecular subtypes (class 1, 2a, 2b, and 3 in UROMOL2021) (32). Anurag et al. showed that CIS samples had a 46-gene expression signature in which the druggable targets *MTOR*, *TYK2*, *AXIN1*, *CTP1B*, *GAK*, and *PIEZO1* were selectively upregulated while *BRD2* and *NDUFB2* were selectively downregulated (107). With stage-stratified multi–*omics* profiling of NMIBC (Ta vs. T1), unsupervised clustering of copy number data revealed four clusters (CN1-CN4) within all tumor samples. Furthermore, Hurst et al. showed that there was sufficient molecular heterogeneity in both stages and, therefore, proposed to divide the Ta and T1 stages into three and four expression groups (TaE1-TaE3 vs. T1E1-T1E4), respectively, which provided prognostic information (108). Strandgaard et al. observed that post-BCG CD8 T-cell exhaustion was associated with post-BCG high-grade (HG) recurrence. They found that pre-BCG tumors of patients with HG recurrence had high expression of genes related to cell division and immune function, and the post-BCG urine of these patients had higher concentrations of immunoinhibitory proteins (CD70, PD1, CD5). A high pre-BCG exhaustion score, calculated based on the mean expression of five immunoinhibitory processes-related genes (*PDCD1*, *CTLA4*, *LAG3*, *HAVCR2*, and *KLGR1*), was associated with worse post-BCG recurrence-free survival (109). Using multiplatform mutational, proteomic, and metabolomic spatial mapping on a whole-organ scale, Czerniak et al. identified the molecular evolution of BC from mucosal field effects, which might span nearly 30 years and can be divided into two phases: The dormant phase was characterized by the gradual development of α mutations. The progressive phase lasted approximately five years and was signified by the β mutations, while the γ mutations developed during the last 2-3 years of disease progression to MIBC (110). By employing mutation, CNA, methylation, mRNA, and lncRNA profiling, Lu et al. refined the consensus classification of MIBC (30) and identified four robust integrative consensus subtypes (iCS1-iCS4) which had distinctive molecular patterns and were associated with stratified prognosis, different tumor immune microenvironment, and distinct sensitivity to immune checkpoint inhibitor therapy (111). Another multi–*omics* approach (mutation, methylation, mRNA, miRNA, and lncRNA) integrated with machine learning revealed three cancer subtypes (CS1-CS3) in MIBC that were related to prognosis and identified 12 hub genes that constituted a consensus machine learning-driven signature (CMLS). The low-CMLS group exhibited a more favorable prognosis and responded better to immunotherapy, while the high-CMLS group had a poor prognosis and a lower likelihood of benefitting from immunotherapy (112). In a recent multi–*omics* study, linoleic acid metabolism was found to be associated with variations in trained immunity induced by distinct BCG strains (113). With proteogenomic characterization, Groeneveld et al. demonstrated five unsupervised proteomic groups (uPG_A-uPG_E) in NMIBC and MIBC. They also identified the enrichment of proteins involved in tumor necrosis factor-related apoptosis-inducing ligand (TRAIL)-mediated apoptosis in FGFR3-mutated tumors, which could not be captured through transcriptomics (114).

Further multi–*omics* studies depicted distinct predictive or prognostic biomarkers for prognosis, programmed death ligand 1 (PD-L1) expression, response to chemotherapy and immunotherapy, immune escape of BC, and immune infiltrates (115–119). A bi*–omics* study revealed that a TME score (low vs. high) can predict the prognosis and the response to immunotherapy (120). Another study defined therapeutic vulnerability gene combinations and prognostic risk of BC by integrating multi*–omics* and clinical data (121). In two other studies, jorunnamycin A and talaroconvolutin-A were found to suppress MIBC via targeting fatty acid synthase (FASN) and topoisomerase 1 (TOP1), and cell cycle and ferroptosis, respectively, which could be a potential candidate for treating BC (122, 123). These existing results can be interpreted as proof that multi*–omics* will provide us a better understanding of BC.

The following impacts can be expected in the management of NMIBC with the success of a multi–*omics* approach:

1. Improvement of the disease outcome of NMIBC by tailoring available treatment options according to the tumor’s molecular profile and predicted treatment response. As such, the proposed approach has a significant direct impact on patient-relevant issues: survival and quality of life.
  - Clinical risk models currently used for intermediate- and high-risk NMIBC patients are based on clinical parameters and lack accuracy. Moreover, they only provide an estimation of the risk of a tumor to recur or progress and offer physicians predictions that are not accurate enough for precision medicine. Therefore, urologists/multidisciplinary teams treat their patients based on ‘subjective’ decisions (interpretation of a certain percentage risk). Incorporating biomarkers into the risk-scoring systems may provide accurate and individual predictions. Thus, through better risk stratification, physicians will avoid over- and under-treatment of NMIBC patients and change the diagnostic and therapeutic decision tree. By improved prediction of the risk of progressing to muscle-invasive disease at the time of initial TURBT, patients who are not likely to respond to conservative treatments (such as intravesical BCG) may be directed to early radical cystectomy in a timely manner and may be offered an increased probability of cure. Better risk prediction may also improve treatment guidelines and lead to more ‘objective’ treatment decisions.
  - Accurate prediction of the risk of progressing to MIBC would also allow the identification of a subgroup of patients in whom conservative treatment modalities may be considered safe. These patients represent the majority of the intermediate- and (very-)high-risk NMIBC group, and accurate risk prediction would allow them to avoid aggressive and invasive treatments (radical cystectomy, chemotherapy, radiotherapy). As a result, their quality of life would be retained. Moreover, for patients in whom the risk of developing future MIBC would be estimated to be elevated by the use of biomarkers, cancer-specific and overall survival may increase as they could be offered early curative treatment.
2. The interactions between putative causative factors, individual features of recurrence and progression, and the spectrum of molecular alterations underlying disease heterogeneity in NMIBC (e.g., response to BCG treatment) will be revealed. In this way, the data would enable the identification of novel therapeutic targets in NMIBC and guiding caregivers in directing the patients who are not responding to gold-standard treatments.
3. If patients suitable for conservative treatment could be determined more accurately and objectively by using biomarkers, the cost of treatment may decrease with a patient-tailored treatment and follow-up scheme. Besides, better risk stratification of NMIBC patients would significantly increase the quality of BC treatment.

The risk stratification developed based on this hypothesis will require external validation. This will prove its scientific accuracy and potential for use in clinical routine practice. In addition to this, it will need valorization in a real-life clinical setting through evaluation of its added value to the quality of treatment and modification of treatment and follow-up, which will impact the cost of disease management and the quality of life of the patients (**Figure 5**).

In conclusion, it is evident that there is ample room for further research to develop a better and more accurate stratification of intermediate- and (very-)high-risk NMIBC. Multi-layered *–omics* studies can provide the ‘missing’ information necessary for increasing the quality of treatment and the quality of life of these patients, as well as for determining novel therapeutic targets.

## Data Availability

The original contributions presented in the study are included in the article/supplementary material, further inquiries can be directed to the corresponding author/s.

## Glossary

4E-BP1: Eukaryotic translation initiation factor 4E (eIF4E)-binding protein 1
ACOT11: Acyl-CoA thioesterase 11
ADGRF1: Adhesion G protein-coupled receptor F1
AK1: Adenylate kinase 1
AKT: RAC(Rho family)-alpha serine/threonine-protein kinase (also known as Protein kinase B)
ALDH1A3: Aldehyde dehydrogenase 1 family member A3
ANXA1: Annexin A1
APC: Adenomatous polyposis coli
AREG: Amphiregulin
ARID1A: AT-rich interactive domain-containing protein 1A
ATM: Ataxia-telangiectasia mutated
ATR: Ataxia-telangiectasia and Rad3-related protein
BAD: BCL2-associated agonist of cell death
BC: Bladder cancer
BCC: Bayesian consensus clustering
BCG: Bacillus Calmette-Guérin
BCL2: B-cell lymphoma 2
BCL2L1: BCL-2-like protein 1
BLVRB: Biliverdin reductase B
BRCA1: Breast cancer type 1 susceptibility protein
BRCA2: Breast cancer type 2 susceptibility protein
BRIP1: BRCA1-interacting protein
C8A: Complement C8 alpha chain
CA3: Carbonic anhydrase 3
CAPG: Macrophage-capping protein
CCA: Canonical component analysis
CCND1: Cyclin D
CD2AP: CD2-associated protein
CDH1: Cadherin-1 (E-cadherin= epithelial cadherin)
CDK4/6: Cyclin-dependent kinase 4/6
CDK12: Cyclin-dependent kinase 12
CDKN1A: Cyclin-dependent kinase inhibitor 1A
CDKN2A: Cyclin-dependent kinase inhibitor 2A
CDKN2B: Cyclin-dependent kinase 4 inhibitor B
circRNA: Circular RNA
CIS: Carcinoma *in situ;* CMLS, Consensus machine learning-driven signature
CKIα: Cyclin-dependent kinase inhibitor α
CNA: Copy number alteration
Co-A: Co-activator
CTLA4: Cytotoxic T-lymphocyte associated protein 4
CTSG: Cathepsin G
CUETO: Club Urologico Español de Tratamiento Oncologico
DAPK: Death-associated protein kinase
DAPK1: Death-associated protein kinase 1
DCCA: Deep cross-omics cycle attention
Deptor: DEP domain-containing mTOR-interacting protein
DDR: DNA damage repair
DBiT-seq: Deterministic barcoding in tissue sequencing
DLL: Delta-like ligand
E2F3: Transcription factor E2F3
EAU: European Association of Urology
EGF: Epidermal growth factor
EGFR: Epidermal growth factor receptor
eIF4B/E: Eukaryotic translation initiation factor 4A/B/E
ELK1: ETS-like 1
EMT: Epithelial-to-mesenchymal transition
EOMES: Eomesodermin
EORTC: European Organization for Research and Treatment of Cancer
EP300: E1A-associated protein p300
ERBB2: Erythroblastosis oncogene B2 (human epidermal growth factor 2=HER2)
ERK1/2: Extracellular signal-regulated kinase ½
ESM1: Endothelial cell-specific molecule 1
FASN: Fatty acid synthase
FAT4: FAT tumor suppressor homolog 4
FF: Fresh frozen
FFPE: Formalin-fixed paraffin-embedded
FGF: Fibroblast growth factor
FGFBP1: Fibroblast growth factor-binding protein 1
FGFR: Fibroblast growth factor receptor
FGFR3: Fibroblast growth factor receptor 3
FHIT: Fragile histidine triad protein (Bis[5’-adenosyl]-triphosphatase)
FOS: Finkel–Biskis–Jinkins murine osteogenic sarcoma virus
FRMD: FERM domain-containing protein
GDF15: Growth differentiation factor 15
GLI1/2/3: Glioma-associated oncogene 1/2/3
GLIFL: Full-length glioma-associated oncogene
GMPS: Guanine monophosphate synthase
GSK3: Glycogen synthase kinase 3
GSTM1: Glutathione S-transferase mu 1
GSTP1: Glutathione S-transferase pi 1
GUCG: Genito-Urinary Cancer Group
HAVCR2: Hepatitis A virus cellular receptor 2
HIC1: Hypermethylated in cancer 1 (ZBTB transcriptional repressor 1)
HG: High grade
HOXA9: Homeobox A9
HPGD: Hydroxyprostaglandin dehydrogenase 15
HSPB1: Heat shock protein family B (small) member 1
IARC: International Agency for Research on Cancer
ICA: Independent component analysis
iCS: Integrative consensus subtypes
IKK: Inhibitor of nuclear factor κ
Indel: Insertion-deletion
ISL1: ISL LIM homeobox 1 (Islet1)
IRPS: Immune-related prognostic signature
JAG: Jagged
KDM6A: Lysine demethylase 6A
KEGG: Kyoto Encyclopedia of Genes and Genomes
KIBRA: Kidney and brain expressed protein
KIF7: Kinesin family member 7
KLRG1: Killer cell lectin-like receptor subfamily G member 1
KMT2C: Lysine methyltransferase 2C
KMT2D: Lysine methyltransferase 2D
KISS1: KiSS-1 metastasis suppressor (kisspeptin)
LAG3: Lymphocyte-activating 3
LATS1/2: Large tumor suppressor kinase 1
LC: Liquid chromatography
LEF: Lymphoid enhancer-binding factor
lncRNA: Long non-coding RNA
LOH: Loss of heterozygosity
LRAcluster: Low rank approximation based multi-omics data clustering
LRP5/6: Low-density lipoprotein receptor-related protein 5/6
MAML: Mastermind-like protein
MAPK: Mitogen-activated protein kinase
Mb: Megabase
MDM2: Mouse double minute 2 homolog
MEK1/2: Mitogen-activated protein kinase 1/2
MER: MER proto-oncogene
MGMT: O-6-methylguanine-DNA methyltransferase
MIBC: Muscle-invasive bladder cancer
miRNA: Micro RNA
MKL: Multiple kernel learning
MLH1: MutL homolog 1
mLST8: mammalian lethal with SEC13 protein 8
MOB1: Mps one binder 1
MOFA+: Multi-omics factor analysis
MONET: Multi-omic clustering by non-exhaustive types
mRNA: Messenger RNA
MS: Mass spectrometry
MSH2: MutS homolog 2
MSH6: MutS homolog 6
MSI: Microsatellite instability
mSIN1: mammalian stress-activated protein kinase interacting protein 1
MSK1: Mitogen- and stress-activated kinase 1
MST1/2: Macrophage-stimulating 1/2
mTOR: Mammalian target of rapamycin
mTORC1/2: Mammalian target of rapamycin complex 1/2
MYB: Myeloblastosis
MYC: Myelocytomatosis
NEMO: Neighborhood-based multi-omics clustering
NFκB: Nuclear factor kappa-light-chain-enhancer of activated B cells
NFKB1: Nuclear factor kappa B subunit 1
NICD: NOTCH intracellular domain
NMIBC: Non-muscle-invasive bladder cancer
NMF: Non-negative matrix factorization
NOTCH: Neurogenic locus notch homolog protein
NPS: NMIBC proteomic subtype
ONECUT2: One cut homeobox 2
PANTHER: Protein analysis through evolutionary relationships
PARADIGM: Pathway recognition algorithm using data integration on genomic models
PCA: Principal component analysis
PCDH8: Protocadherin 8
PCDH17: Protocadherin 17
PCDHGA12: Protocadherin gamma subfamily A, 12
PDCD1: Programmed cell death 1
PDK1: Protein 3-phosphoinositide-dependent protein kinase-1
PD-L1: Programmed death ligand 1
PGAM1: Phosphoglycerate mutase 1
PI3K: Phosphoinositide 3-kinase
PIK3CA: Phosphatidylinositol-4,5-biphosphanate 3-kinase, catalytic subunit alpha
PIP3: Phosphatidylinositol (3,4,5)-triphosphate
PLCγ: Phospholipase C gamma
PIntMF: Penalized integrative matrix factorization
PMF1: Polyamide modulated factor 1
POLD1: DNA polymerase delta 1, catalytic subunit
POLE: DNA polymerase epsilon, catalytic subunit
POLQ: DNA polymerase theta
POU4F2: POU class 4 homeobox 2
PRAC1: PRAC1 small nuclear protein
PRAS40: Proline-rich AKT1 substrate
PRDX1: Peroxiredoxin 1
PRDX2: Peroxiredoxin 2
PRDX6: Peroxiredoxin 6
Protor: Protein observed with Rictor
PSME1: Proteasome activator subunit 1
PTCH1: Patched 1
PTEN: Phosphatase and tensin homolog
PTGDR: Prostaglandin D2 receptor
PVRL4: Poliovirus-receptor-like 4 (Nectin 4=Nectin cell adhesion molecule 4)
RAF: Rapidly accelerated fibrosarcoma
Raptor: Regulatory-associated protein of mTOR
RAS: Rat sarcoma
RASSF1: Ras-associated domain family member 1
RASSF1A: Ras association domain-containing protein 1A
RB: Retinoblastoma protein
RB1: RB transcriptional corepressor 1
Rictor: Rapamycin-insensitive companion of mTOR
RPB-J: Recombination signal binding protein for immunoglobulin kappa J region
RUNX3: RUNX family transcription factor 3
S6K1: Ribosomal protein S6 kinase beta-1
SALMON: Survival analysis learning with multi-omics neural networks
SAV1: Protein Salvador homolog 1
scAI: Single-cell aggregation and inference scMVAE, Single-cell multimodal variational autoencoder
SFRP5: Secreted frizzled-related protein 5
SHH: Sonic hedgehog
SMAD1-4: Mothers against decapentaplegic homolog 1-4
SMO: Smoothened
SNF: Similarity network fusion
SNRPD2: Small nuclear ribonucleoprotein D2 polypeptide
SNV: Single nucleotide variation
SOCS1: Suppressor of cytokine signaling 1
SPR: Sepiapterin reductase
SUFU: Suppressor of fused kinase
SUMO: Small ubiquitin-like modifier
SWI/SNF: SWItch/Sucrose Non-Fermentable
STAT3: Signal transducer and activator of transcription 3
TAP1: Transporter 1, ATP binding cassette subfamily B member
TAZ: Transcriptional coactivator with PDZ-binding motif
TBX3: T-box transcription factor 3
TBX4: T-box transcription factor 4
TCF: T-cell factor
TCGA: The Cancer Genome Atlas
TEAD1-4: Telomere ends-associated domain family member 1-4
TGF-α: Transforming growth factor-alpha
TGFB1: Transforming growth factor beta 1
TIMP3: TIMP metallopeptidase inhibitor 3
TLR: Toll-like receptor
TMB: Tumor mutational burden
TME: Tumor microenvironment
TOP1: Topoisomerase 1
TP53: Tumor protein 53
TRAIL: Tumor necrosis factor-related apoptosis-inducing ligand
TRANSFAC: Transcription Factor database
TRK: Tyrosine receptor kinase
TSC1/2: Tuberous sclerosis 1/2
t-SNE: T-distributed stochastic neighbor embedding
TURBT: Transurethral resection of the bladder tumor
TWIST1: Twist-related protein 1
UC: Urothelial carcinoma
UMAP: Uniform manifold approximation and projection
UMP/CMPK: Cytidine/uridine monophosphate kinase 1
uPG: Unsupervised proteomic group
VEGF: Vascular endothelial factor
VEGFR: Vascular endothelial factor receptor
VHL: Von Hippel-Lindau tumor suppressor
WFDC2: WAP four-disulfide core domain protein 2 (human epididymis protein 4)
WHO: World Health Organization
WIF1: Wnt inhibitory factor 1
Wnt: Wingless-Int 1
YAP: Yes-associated protein
ZNF154: Zinc finger protein 154
